# Supplementary material for: Are carbon emissions trading and green financial instruments synergistic? -Comprehensive quantitative research based on content analysis
Source: PLoS One. 2024 Mar 7;19(3):e0298601. doi: 10.1371/journal.pone.0298601 (PMC10919601; doi:10.1371/journal.pone.0298601)
Supplement: S1 Data — (ZIP) [file pone.0298601.s001.zip › Data/地级市Z_F工作报告.zip!/░▓╗╒/░÷▓║╩╨/└┤╫╘╣1⁄2╓┌║┼í╛┬φ┐╦╩2╛▌═°í┐_2013.docx]

各位代表：
   现在，我代表市人民政府向大会报告工作，请予审议，并请市政协委员和其他列席人员提出意见。
   一、回顾过去，经济社会发展跃上了新台阶
   刚刚过去的2012年，是实施“十二五”规划承上启下的重要一年，也是本届政府的收官之年。一年来，面对严峻复杂的宏观经济形势，全市上下在市委的坚强领导下，深入贯彻落实科学发展观，牢牢把握稳中求进、持续快进的总基调，全力以赴稳增长、调结构、抓创新、惠民生、促和谐，主要经济指标逆势上扬，总体形势超出预期、好于全省，较好地完成了市十四届人大六次会议确定的各项目标任务。预计，全年完成地区生产总值890亿元，同比增长13%左右，增速位居全省前列。其中，一、二、三产增加值分别增长5.5%、16.5%、11.5%。财政收入164.7亿元，增长21.1%，其中地方收入78.4亿元，增长27.8%。固定资产投资810亿元，增长27%。社会消费品零售总额372亿元，增长16.7%。城镇居民人均可支配收入20685元，增长14%，突破2万元大关。农民人均纯收入7810元，增长18%。万元地区生产总值综合能耗、主要污染物减排持续下降。
   (一)不断优化产业结构，经济持续快速发展
   工业经济强势增长。强化运行调度和政策引导扶持，启动实施“服务企业百日提升行动”，新增规模以上企业145户，实现规模以上工业增加值390亿元，增长18%以上。工业企业实现利润44亿元，增长18%。工业化率达45%左右。安瑞科大型压缩机制造基地、华芳纺织一期等70个项目建成投产，大富机电射频器件二期、晟光科技电容式触摸屏二期等73个项目开工建设，总投资44.6亿元的国电蚌埠电厂二期工程成功获批。皖酒品牌被评为中国驰名商标。
   农业农村经济稳步提升。粮食总产达261万吨，实现“九连增”。流转土地60万亩，新增标准化设施蔬菜面积1.1万亩、花卉苗木基地1万亩，规模养殖比重提高2个百分点。新增规模以上农产品加工企业55家。组建农民专业合作组织299个。深入开展“农田水利建设高潮年”活动，新增旱涝保收田14.5万亩。成片造林5.82万亩，新建绿色长廊85公里。连续14年实现耕地占补平衡。
   服务业发展亮点纷呈。深入开展全国农产品现代流通综合试点工作，成功列入全国中小商贸流通企业服务体系、家政服务试点城市。新增限额以上商贸企业198户。南山豪生大酒店、金润国际广场一期基本建成，星宇文化产业园、义乌国际商贸城、蚌埠国际汽车城等项目开工建设。花鼓灯嘉年华一期、森禾现代花卉科技产业园建成运营，旅游市场日趋繁荣。
   县域和开发区经济竞相发展。积极实施县域经济腾飞计划，三县地区生产总值增长15%左右，主要经济指标增速均超过省、市平均水平。开发区加速集聚，省级以上开发区完成工业总产值突破1000亿元，增长35%，占全市工业总产值的63%。
   (二)深入推进城乡建设，承载能力有效提升
   城市大建设步伐加快。实施项目117个，完成投资170亿元。市区完成征迁拆违520万平方米，交付净地2.6万亩。开工建设各类安置房310万平方米，竣工200万平方米。“十大工程”顺利推进，东海大道贯通提升、中环线高新区段建成通车，市民广场、博物馆、档案馆、规划馆、综合客运站等加快建设。大庆路淮河公路桥、城市西出口、解放路南段一期等38个项目竣工交付。合蚌客运专线正式运营，民航机场启用前期工作有序开展。《蚌埠市规划人口及建设用地规模专题研究》获得批准，核定到2030年中心城区人口规模为220万人，用地规模为220平方公里，位居全省前三、皖北第一。
   城乡人居环境持续改善。集中整治十大环境突出问题，八里沟、张公湖、鲍家沟区域环境整治等取得明显成效。新建雨污管网50公里，杨台子污水处理厂二期建成试运营。完成东海大道、燕山路等主干道绿化提升工程，新建、改造街头游园绿地23个。推进环卫、绿化责任重心下移，完善城市网格化管理。全面开展美好乡村建设，完成中心村布点，启动首批中心村规划编制和17个示范中心村建设工作，怀远县4个试点村工程建成投用。完成农村公路县乡道升级改造500公里，危桥改造200座，基本实现“村村通”。
   (三)着力扩大有效投入，发展后劲切实增强
   投资规模不断壮大。深入实施“3461”行动计划，完成投资460亿元。现代牧业一期、昊锐太阳能背板一期等88个项目建成投产，万达广场、特步鞋服生产基地、蚌埠玻璃设计院超薄电子基板等265个项目加快推进，安徽冠宜箱包制造基地、和鼎半钢子午轮胎等175个项目开工建设。争取中央、省补助资金12.1亿元，净增1.5亿元。
   招商引资势头强劲。扎实开展“招大引强攻新年”活动，成功举办“一节三会”。引进亿元以上项目110个，台嘉玻璃电子级玻璃纤维、北方通用电子MEMS产业基地等16个10亿元以上项目落户我市。实际利用市外资金668亿元、增长30.4%，利用外商直接投资6.6亿美元、增长44.8%。
   金融创新和融资能力明显提升。新增贷款151亿元，增长31%，增量存贷比达122%，增速和增量存贷比均居全省第1位。“珠城创新”中小企业集合信托二期成功发行。光大银行落户我市，市区农信社成功改制为蚌埠农村商业银行，怀远本富村镇银行、银河证券蚌埠营业部挂牌运营。新增小额贷款公司3家、融资租赁公司2家、融资性担保公司3家，率先在全省试点小额贷款保证保险。组建了水投公司，城投、蚌投、交投等融资能力进一步增强。
   (四)积极深化改革创新，转型升级步伐加快
   战略性新兴产业集聚发展。硅基功能材料列入国家战略性新兴产业集聚发展试点取得积极进展，电子信息首位产业集群集聚效应初显，高端装备制造、生物医药、新材料、节能环保、新能源等产业统筹推进。华益电容触摸屏用导电膜玻璃、新源安徽环保装备产业基地一期等项目建成投产，盛世光电太阳能电池用超白基板、鑫源石英材料软硅生产等项目开工建设。战略性新兴企业发展到93家，实现产值320亿元，增长30%。
   自主创新试验区建设不断深入。成功创建国家科技和金融结合、国家知识产权质押融资试点市。专利权质押贷款规模全省第一，商标专用权质押贷款突破2亿元。成功组建蚌埠电子信息、硅基新材料产业技术研究院，新建省级以上研发平台18家，新增高新技术企业28家、省级创新型企业8家。中电科41所、40所整合业务资源，合作成立专业化公司，加快民用产品开发。发明专利授权量280件，居全省第3位。合芜蚌(蚌埠)人才金港开港，11家企业列入股权和分红激励试点范围。
   改革开放稳步推进。基本药物制度实现全覆盖。全面实施县级公立医院综合改革，药价降幅35%以上。完成事业单位清理规范工作，累计撤销、合并49家单位。交通运输业和部分现代服务业营改增试点顺利开展，市级预算单位公务卡制度全面推行。连续两年被评为全国文化体制改革先进地区。新增私营企业2740家、个体工商户16038户。成功列入中原经济区规划。口岸建设不断加强，铁海联运专列顺利开通。完善促进外贸发展政策，实现进出口总额12亿美元，增长55%。
   (五)致力保障改善民生，社会大局和谐稳定
   民生工程深入实施。投入资金41亿元，增长17.1%，全面完成40项省、市民生工程。大力推进“孔雀计划”，城镇新增就业6.2万人，城镇登记失业率3.2%。在全省率先建立多层次医疗保障体系，城乡居民社会养老保险实现全覆盖。发放城乡低保金2.84亿元。开工建设保障性住房8934套，实物配租2659户，发放租金补贴15466户。建设集中供水厂12处，解决了17.7万人农村饮水不安全问题。乡镇文化站和农家书屋建设全面完成。资助贫困大学生987名，补助“一户多残”家庭1800户，成功创建示范社区8个。
   社会事业协调发展。投入义务教育保障经费2.74亿元，惠及33.8万名学生。新改扩建幼儿园46所，二中新校区主体封顶。进城务工人员随迁子女实现“零障碍”入学。蚌医一附院新门急诊和综合病房楼投入使用，市疾控中心、中医院新院区主体竣工，皖北药品检验中心开工建设。禹会村遗址、汤和墓等7处文物单位被确定为全国文物保护单位。体育场东看台改造和全民健身活动中心基本建成。投入1.6亿元，新购置公交车385辆。实现全国双拥模范城“五连冠”。新当选“中国好人”12人，总量位列全省第二位。人口和计划生育、安全生产、民族宗教、外事、侨务、地方志、审计、统计、公积金管理等工作得到加强，国防动员、人防、档案、地震、气象、妇女、儿童、老龄和残疾人等事业取得新进展。
   社会管理切实加强。推进社区管理体制改革试点，撤销街道办事处6个。科技化治安防控体系不断完善，连续6年获评省综治先进市。妥善处置突发事件，医患纠纷第三方调处机制“蚌埠经验”得到中央、省领导批示肯定，在全省总结推广。认真落实社会稳定风险评估机制，深入开展大接访、大走访活动，积极化解一批信访积案，实现十八大期间进京上访“零”记录，社会大局和谐稳定。
   (六)促进机关效能提升，自身建设不断强化
   深化行政审批制度改革，审批效能持续提升。开展招投标市场公共服务体系试点，《公共资源交易市场服务标准体系》被确定为安徽省地方标准。自觉接受人大、政协监督，办理市人大代表议案6件、建议90件，市政协提案316件。加强党政门户网站建设，政府信息公开水平稳步提高。首次评选工作创新奖。创新市长热线运行机制，设立政府服务企业热线，拓宽为民服务新渠道。扎实推进廉政风险防控工作，严肃查处一批违纪违法案件，发展环境进一步优化。
   各位代表，2012年目标任务的全面完成，为本届政府五年来的工作划上了圆满的句号。
   这五年，是老工业基地复苏振兴，综合实力大幅提升的五年。地区生产总值年均增长13.2%，顺利实现翻番目标。规模以上工业增加值年均增长21.1%，工业化率提高近10个百分点，二三产业比重达81.7%。财政收入、固定资产投资分别是2007年的2.9倍、4.1倍。经济增长的稳定性、协调性和可持续性逐步增强。
   这五年，是自主创新试验区建设深入推进，改革开放取得突破的五年。高新技术产业增加值增长3倍，高新技术企业、科技创新平台、专利授权量等指标跃居全省三甲，成功跻身国家科技进步先进市。高新区晋升为国家级，省级以上开发区达8家。投融资、文化、医药卫生等重点领域改革深入推进。进出口总额增长2.7倍。累计引进市外资金增长4.8倍，中建材、中粮、中纺、特步、台玻等一批知名企业落户蚌埠。
   这五年，是区域性中心城市地位巩固提升，城乡面貌发生巨大变化的五年。京沪高铁蚌埠南站投入使用，合蚌客专、蚌淮高速建成通车，交通枢纽地位进一步凸显。大力开展城市大建设，滨湖新区、高铁新区、城南新区雏形初现，城市框架持续拉开，市区建成区面积111.5平方公里、常住人口110万人。县城综合承载力有效提升，农村基础设施积极改善，市县联动发展格局初步形成。城镇化率提高7.5个百分点，达48.2%。
   这五年，是人民生活水平明显提高，社会建设全面加强的五年。城乡居民收入年均分别增长12.8%、16.7%。累计投入资金113亿元，共实施48项省、市民生工程。新增就业22.2万人，社会保障能力显著增强。各类各级教育事业加快发展，文化事业不断繁荣，城乡公共卫生体系继续完善，全民健身活动蓬勃开展。城镇居民居住条件逐步改善，人均住房建筑面积由26平方米增加到32平方米。
   各位代表，五年的开拓奋进，五年的辉煌成就，奠定了科学发展、全面转型的坚实基础，开启了加速崛起、富民强市的崭新局面。经济社会发展取得的每一项成就、城市发生的每一处变化、百姓得到的每一笔实惠，无不凝聚着全市劳动者的心血与汗水，无不饱含着方方面面的关心与支持。在此，我谨代表市人民政府，向努力工作在各行各业的广大工人、农民、知识分子、干部和解放军指战员、武警官兵、政法干警，向给予政府工作大力支持的人大代表、政协委员，向国家和省驻蚌单位，向各民主党派、工商联、各人民团体和其他各界人士，向在蚌创业的境内外投资者和建设者，向所有关心、支持和参与蚌埠改革发展的同志们、朋友们，向为蚌埠发展做出过突出贡献的历届历任老领导、老同志，表示衷心的感谢和崇高的敬意！
   回顾过去五年，成绩有目共睹，经验弥足珍贵，同时问题也客观存在。主要表现在：经济总量还不够大，位次仍处于全省中游水平，争先进位的任务艰巨；产业结构还不够优，工业化水平较低，开发区支撑作用不足，提高优势产业核心竞争力和新兴产业带动力的任务艰巨；城市的承载功能还不够强，品位还不够高，巩固提升中心城市地位的任务艰巨；农业发展方式转变还不够快，农业科技和装备水平还不高，农村基础设施还比较薄弱，一二级公路占比较低，统筹城乡发展的任务艰巨；水资源保护利用力度不够，林业发展水平特别是森林覆盖率不高，环境保护和生态建设的任务艰巨；社会事业发展不足，公交、公园、公厕等公共服务设施薄弱，保障和改善民生的任务艰巨；政府职能转变还不到位，创新体制机制、优化发展环境的任务艰巨。面对这么多的艰巨任务，我们将高度重视，认真分析，研究路线图，制订时间表，鼓足勇气攻坚克难，只争朝夕推动发展。
   二、展望未来，倾力打造全面崛起的新蚌埠
   各位代表，成绩属于过去，奋斗成就未来。蚌埠正处在黄金机遇期、城市扩张集聚期和经济加速发展期。未来五年，尽管我们还会遇到很多困难和问题，尤其是面临区域竞争和要素约束的双重压力，但我们坚信机遇大于挑战，只要科学谋划、提振信心，就能无往不前。国家实施中部崛起战略、推进中原经济区建设，省委、省政府加快皖北地区振兴步伐、支持区域性中心城市建设，合芜蚌自主创新试验区升级为国家战略，各种政策叠加效应正在集中释放。同时，经过多年的改革发展，我市综合实力明显增强，经济运行中的积极因素加快集聚，跨越发展、加速崛起的势头日趋强劲。尤其是全市上下政通人和、精神振奋，想发展、谋发展、会发展的强大动力持续加大，累积效应日益显现。只要我们紧紧抓住并切实用好各种机遇和有利条件，不为过去成绩所满，不为既有经验所限，不为艰难险阻所惧，奋起争先，昂扬前进，就一定能构筑新优势，再创新辉煌，实现全省上下认可、全市干群认同的全面崛起，成为带动皖北振兴的领头军、安徽加速崛起的重要增长极。
新一届政府工作总体要求是：全面贯彻落实党的十八大精神，以邓小平理论、“三个代表”重要思想、科学发展观为指导，坚持创新驱动，四化同步，大力推进工业强市、农业稳市、商贸活市、科教兴市、文化立市、生态靓市，展示更大手笔，实施更大项目，培育更大产业，推进更大征迁，推动更大建设，改善更大民生，赢得更大发展，主要经济指标增速持续高于全省平均水平，努力建设经济发达、文化繁荣、生态优美、温馨和谐的区域性特大中心城市，重振雄风，再创辉煌，为重返全省第一方阵奠定坚实基础。
   新一届政府工作的主要目标和任务是：
   (一)加快结构优化升级，夯实经济增长基础。大力实施“5116”重点产业发展工程，改造提升精细化工、纺织服装、高质烟酒等传统加工业，培育壮大电子信息、高端装备制造、生物、新能源及新材料等先进制造业，发展繁荣彰显蚌埠特色的文化旅游业，着力攻坚现代物流、新型商贸、金融等高端服务业，积极发展现代农产品种养、食品加工等现代大农业，力争重点产业增加值突破1300亿元，产值过100亿元企业达10家。深入实施工业强市战略，推动骨干企业和优势产业集聚集约发展，打造电子信息、装备制造2条千亿产业链，工业化率提高7个百分点，二三产业增加值占生产总值比重达87%以上。完成固定资产投资6500亿元，推动地区生产总值、财政收入翻番，主要经济指标在省内增速保前列、总量进位次。
   (二)加快城市建设步伐，打造区域性特大中心城市。围绕2030年远期目标，协调推进新城区建设和老城区改造，推动各功能区高水平开发，加快与怀远县城等周边城镇同城化发展步伐，构建大蚌埠都市区，力争到2017年市区建成区面积突破150平方公里、常住人口达150万。按照“引河入城、拥河发展、环湖集聚、显山露水”的思路，构筑一心多极的空间结构和“一河、三湖、多廊道”的山水格局。建设城市中环线、高速外环线，新增跨河通道，实施水运振兴工程，开通民用航线，完善立体式综合交通体系。坚持拆迁、规划、建设、绿化、管理“五位一体”，继续开展城市大建设。加大水环境整治和活水改造力度，让灵动之水跃动魅力珠城。加强环境保护和生态建设，争创国家级生态市和园林城市，努力建设天蓝、地绿、水净的生态蚌埠、美丽蚌埠。
   (三)加快四化同步进程，推动城乡一体化发展。推动信息化和工业化深度融合、工业化和城镇化良性互动、城镇化和农业现代化相互协调，推进公共资源均衡配置、生产要素自由流动，促进城乡共同繁荣、一体发展。科学编制全地域规划，鼓励支持怀远、固镇、五河县城向中等城市迈进，加快建设扩权强镇试点镇和中心镇，全面推进美好乡村建设，实现城乡基础设施共建共享、产业发展互促互动，着力构建以市区为核心、县城为骨干、乡镇为节点、中心村为补充的新型城镇化发展格局。加快天河湖、四方湖、芡河等区域基础设施建设，加大资源整合开发力度，打造西部山水文化、生态旅游景区。引导人口向城镇聚集，推动农民向市民转变，城镇化率达55%以上。
   (四)加快推进改革开放，提升区域发展竞争力。注重顶层设计，强化政策引导，深入推进自主创新综合试验区建设，积极构建独具蚌埠特色的创新体系。加快科技创新步伐，新增高新技术企业100家，努力打造科技市、创新城。推进行政职能转变，加强行政管理创新，稳步实施教育、文化、医药卫生、收入分配等重点领域和关键环节改革。推动省级以上开发区扩区增容、转型升级，力争建成区面积扩大80平方公里、工业总产值达2600亿元。积极参与中原经济区、泛长三角区域发展分工与合作，引进和实施一批龙头型、旗舰型、领军型项目，累计利用省外亿元以上项目资金3000亿元，年均增长20%以上，努力成为引资聚智的焦点、承东启西的节点、南来北往的亮点。
   (五)加快打造文化强市，突出文化引领作用。大兴文化建设，提高文化软实力，发挥文化引领风尚、教育人民、服务社会、推动发展的作用。加快文化体制改革，促进文化事业和文化产业协调发展。完善公共文化服务设施，不断满足人民群众日益增长的精神文化需求。充分挖掘、宣传大禹、涂山、双墩、垓下等文物遗迹的历史价值，彰显独具蚌埠特色的文化内涵和巨大影响力。大力发展文化创意、数字动漫、影视传媒等文化产业，打造一批竞争力强的骨干企业和文化品牌，增强蚌埠特色文化的时代感和生命力。加强社会主义核心价值体系建设，不断提升市民文明素质和道德修养，积极创建全国文明城市提名城市。
   (六)加快社会民生建设，营造温馨和谐的社会环境。坚持以人为本，着力推动解困民生向普惠民生发展，向幸福民生攀升。深入实施城乡居民收入倍增规划，逐步缩小收入差距。扎实推进省级各项民生工程，每年实施一批群众需求急、实惠大的市级民生工程。大力支持敬老院、幼儿园等与群众生活息息相关的公共服务设施建设，积极构建终身教育、社会保障、就业服务、医疗卫生、住房保障、社会养老服务六大体系，让城乡居民学有优教、劳有多得、住有宜居、病有良医、老有善养。深入推进法治蚌埠建设，加强和创新社会管理，打造温馨蚌埠，切实维护和谐稳定，使全市人民的生活过得更加殷实、更加幸福。
   各位代表，未来五年我们还将遇到一些难以预见的机遇和挑战，还将面对许多省委、省政府的新要求和广大市民的新期待。对此，我们将及时补充、完善各项工作部署，始终坚定加速崛起的信心和决心，切实增强争先进位的紧迫感，咬定目标不动摇，只争朝夕不懈怠，不断创造新业绩，真正让人们感到“走千走万，还是淮河两岸”！
   三、确保今年，奋力开启跨越赶超的新征程
   2013年是全面贯彻落实十八大精神的开局之年，是实施“十二五”规划承前启后的关键一年，也是新一届政府的届首之年，各项工作都要力争开好头、起好步。主要预期目标是：地区生产总值增长12%以上，力争总量突破1000亿元；一、二、三产分别增长3.5%、16.5%和10.5%，规模以上工业增加值增长18%；财政收入增长15%左右，为迈上200亿元台阶打下坚实基础；固定资产投资增长25%；社会消费品零售总额增长15.5%；进出口总额增长25%；城镇居民人均可支配收入和农民人均纯收入分别增长14%、15%；城镇登记失业率、人口出生率、居民消费价格涨幅、单位生产总值能耗、国家要求削减的主要污染物排放均控制在省下达的目标以内。主要经济指标增速“力争高于去年、确保高于全省”，为全面建成小康社会、重返全省第一方阵吹响冲锋号、打胜第一仗。
   实现上述目标，必须重点抓好以下九方面的工作：
   (一)做强产业、企业、园区，推动工业提速增效。坚持扩张总量与调整结构并举，发展速度与质量效益并重，培育发展新兴产业与改造提升传统产业齐抓，加快新型工业化步伐。
   做大做强先进制造业。紧抓领军企业、重大项目、产业链条、产业集聚四大环节，强力推动电子信息、高端装备制造、生物、新能源及新材料四大先进制造业规模化、品牌化、集聚化发展。争取硅基功能材料列入国家级试点，开工建设台嘉玻璃电子级玻璃纤维、中电科机电产业园等项目，推进北方通用电子MEMS产业基地、德豪润达LED芯片封装及产业化等项目，建成投产蚌埠玻璃设计院超薄电子基板、晟光科技电容式触摸屏二期等项目，加快打造千亿级电子信息首位产业。全力推进安徽环保装备产业基地、智能防爆离心机、顺酐及可降解塑料产业化等项目，促进高端装备制造等产业发展。
   改造提升传统优势产业。坚持工业化、信息化、自动化、智能化，采用先进适用技术和信息技术改造提升传统加工业，继续调整优化精细化工产业，巩固提升纺织服装、食品加工、高质烟酒等优势产业。建成安徽中烟烟草薄片、华芳纺织二期、特步鞋服生产基地一期等项目，开工建设中粮粮食产业园、德翔中高密度板等项目。力争国电蚌埠电厂二期形成实物投资。推进信息技术与传统产业融合，新增2家信息化服务平台、3家两化融合示范企业。
实施造舰创牌行动。加强制度、管理、技术、文化创新，增强企业核心竞争力。扶持实体经济发展，支持丰原集团等重点企业做优做强，推进跨地区兼并重组、境外并购和投资合作，新增产值10亿元以上企业3家，力争中粮生化实现产值过百亿目标。大力实施品牌发展战略，争创10个省级以上品牌。支持中电科41所、40所合作公司发展，加快建设电子仪器仪表产业基地。实施“中小企业成长工程”，培育一批专精特新和成长型中小企业。新增规模以上工业企业100户以上。
   统筹推进园区发展。加快机制和管理创新，进一步挖掘潜力、激发活力，推动产城一体，促进开发区转型发展、优化升级，使之成为经济增长的发动机、城市发展的增长极。激发高新区发展活力，力争主要经济指标增速位居省内同级高新区前列。扩大国家级、省级开发区建成区面积，推进蚌埠铜陵现代产业园起步区建设，引导其他省级开发区做大做强。加快电子信息、节能环保、柳工机械等专业园区建设，完成循环产业园前期工作，推动产业集聚。探索园区产业供地和企业进退机制，提高园区土地节约集约利用水平。
   (二)突出商贸、物流、旅游，实现服务业提档升级。坚持市场化、产业化和社会化方向，建项目、强集聚、增规模、优结构，推动服务业突破性发展。
   着力提升商贸等生活性服务业。加快义乌国际商贸城、万达广场、天湖国际城等项目建设，建成投运蚌埠国际汽车城一期、钱江商业中心。新增限额以上商贸企业150户、限额以上服务业企业60户。引进新企业、新业态，激活淮河文化广场商圈。推进全国中小商贸流通企业服务体系和家政服务体系试点建设，新扩建社区服务中心(站)30个，建成社区商业中心3个。稳步发展房地产业，继续抓好保障性住房和普通商品房建设。
   培育壮大物流等生产性服务业。调整优化物流业布局，推进公铁水多式联运，重点发展散装粮食、烟草、冷链等专业物流，打造区域性物流中心。加快淮河新港物流、力源码头等一批物流产业集聚区建设，深化长淮卫临港产业园前期工作。引导发展科技研发、技术推广、工业设计、软件开发等行业，鼓励发展法律、会计、咨询、会展等商务服务业。
   加速发展旅游等现代服务业。抓好景区景点建设，高标准规划启动涂山风景区保护和开发，加快建设花鼓灯嘉年华二期、湖上升明月、大明文化产业园。着力实施喜来登大酒店、迎宾馆等项目，建成鼎丰国际大酒店，提升旅游接待能力。加强品牌和标准化建设，鼓励发展乡村旅游、红色旅游等特色旅游，大力培育4A级景区、星级农家乐。积极发展信息、电子商务、总部经济、服务外包等现代服务业。
   (三)聚力县域、农业、乡村，推进城乡统筹发展。以四化同步为引领，大力实行重点设施向县域延伸、重大产业向县域布局、重要项目向县域倾斜，促进县域和农业农村经济加快发展。
   强力推动县域经济腾飞。统筹产业、项目和人才、资金，促进资源要素向县域流动，确保三县主要经济指标增速持续超全省、全市平均水平，在全省位次不断前移。重点扶持各县发展1-2个优势产业，建成大富机电射频器件二期、中纺油脂蚌埠产业园一期、丰原维生素系列产品等一批重大项目，三县工业投资增长40%以上，规模以上工业增加值增长30%以上。按照30万人口、30平方公里的标准规划建设，拉开发展框架，加快推进三县县城向中等城市迈进。坚持统筹规划、规模适度原则，每个县围绕交通枢纽型、人口集聚型、文化底蕴型、产业特色型规划建设2-3个中心镇，打造统筹城乡发展的重要节点。积极推进市县规划衔接、城乡基础设施对接、城乡公共服务共享，有序引导人口、产业向县城和中心镇集中。注重城乡路网统筹规划、协调发展，完成一二级公路升级改造65.7公里。加强农村建设用地整治，启动“人地挂钩”政策试点，增强用地保障能力。
   大力发展现代农业。实施小麦千斤乡镇、千斤县工程，打造10个万亩高产示范片，力争粮食总产“十连增”。流转土地40万亩，推进农业规模化、产业化、品牌化发展，打造一批现代农业万亩田、万亩场、万亩塘，建设高标准花卉苗木基地1万亩，培育“一村一品”特色专业村。加快农业产业化发展，争创1个国家级农业产业化示范基地、1个国家级现代农业示范区。大力实施蚌埠农业“百佳工程”，创建和表彰十佳万亩土地流转示范片、十佳农业产业化龙头企业、十佳农机专业合作社、十佳美好乡村、十佳农资供应商、十佳农超对接社区“放心店”、 十佳特色产品品牌、十佳种养能手、十佳农技员、十佳乡村物业管理员。加强农田水利建设，实施塘坝扩挖、沟河清淤和小型泵站更新改造，新增旱涝保收田8万亩。
   着力三提狠抓五统。提升科技水平，注重农技人员和农民素质培养，促进农业科研成果和先进适用技术推广应用；提高组织程度，发展农民专业合作社和农业产业化经营；提振产品品牌，县区联手打造一批特色大品牌。统一协调，合力支持新型城镇化与农业产业化齐头并进；统一规划，引导和扶持各类农业经营主体规模发展区域特色产业；统一建设，坚持高起点规划、高标准建设产业园区和美好乡村；统一标准，制定完善和普及应用全市种植业、养殖业、加工业生产标准，提升农产品科技含量和整体竞争优势；统一服务，实行农技人员包村联户责任制，开展从品种选择、种养生产到产品销售的全程服务，统一标识、挂牌上岗。
   合力建设美好乡村。多方参与，多元投入，启动首批129个中心村建设，重点培育17个可看可学、示范带动的样板村，完成第二批110个中心村建设规划编制。创新农村管理机制，开展新型农村社区化建设和物业化管理试点。推进“新网工程”建设，推行农批农超对接和直采配送等营销模式，建设一批农村商品连锁店。大力开展与农民生产生活密切相关的垃圾污水处理、安全饮水、卫生改厕、河塘沟渠清淤等农村环境综合整治工作，引导“改厕、改圈、改厨”，解决“上气、下水、循环”难题，推进人畜粪便、农作物秸秆、生活垃圾和污水的资源化利用，转化生产肥料、燃料、饲料，让广大群众“走平坦路、喝干净水、上卫生厕、住清洁房”，实现生产、生活、生态的三生良性循环。
   (四)强化投资、引资、融资，增强经济发展后劲。坚持把扩大投资与招商引资、搞活融资结合起来，把促进即期增长与支撑长远发展结合起来，千方百计增加有效投入，为蚌埠加速崛起注入强劲动力。
   着力加快重点项目建设。坚持实施重点项目旬报告、月调度、季通报和重大项目市领导牵头联系制度，建立分层分类、逐个调度机制，综合利用信息手段，构建协调联动、实时监控、及时响应的新型项目管理体系。建成蚌固一级公路、银锐玻璃切割设备等项目，加快建设朗润导电纤维、百特纳米级防火保温新材料等项目，开工建设蚌埠玻璃设计院8.5代TFT超薄玻璃基板、方兴科技高纯超细氧化锆、蚌埠-浮山段行蓄洪区调整与建设工程等项目。加大项目谋划力度，新增储备项目规模达1000亿元以上。
   全面提升招商引资水平。继续招大引强攻新，密切关注高新技术、新兴产业前沿动态，着力拓展与央企、省企、知名民企合作空间，开展电子信息产业对接会、软件动漫产业推介会等一批专题招商活动，积极对接华硕电脑、中电电气等一批战略投资者，引进华威光电产业园、巨人网络科技、电动车及配件生产等一批重大项目，加强与新西兰畜牧养殖企业合作。突出京沪高铁沿线、沿海、港澳台及周边国家等重点区域招商，着力引进高端产品、龙头企业、外资项目。实行招商引资市级统筹，引导有序竞争，加快产业集聚。完善招商绩效管理办法，注重后续政策制定和扶持措施预响应，提高项目开办率、开工率和开业率，全年引进超亿元项目100个以上，实际利用市外亿元以上项目资金500亿元、外商直接投资7.6亿美元。三县和高新区、经济开发区确保各新引进2个，四区至少各新引进1个10亿元以上项目。
   持续扩大融资规模。加大信贷投放，新增贷款150亿元，贷款余额770亿元。发行城投公司20亿元二期债券、高投集团8亿元企业债、蚌投集团8亿元短期融资券。按照资本最大化、资产最优化原则，充实蚌投、城投和交投、水投、城开等融资平台资本金，组建市担保集团，大胆、大力、大规模融资。加强与省开行、进出口银行合作，加快固镇、五河农信社改制步伐，引进1家股份制商业银行，组建1家村镇银行。科学规划融资规模和偿还进度，优化金融生态环境，实现经济金融良性互动、持续发展。
   (五)加强规划、建设、管理，做大做优中心城市。突出滨水、生态、文化、服务等特色要素，坚持精品规划、精致建设、精细管理，着力扩大规模、优化布局、完善功能、提升品位，增强中心城市集聚辐射能力。
抓紧完善城市发展规划。拓展思路，丰富内涵，树立全域蚌埠规划理念，严格落实“四规合一”。完成《蚌埠市城市总体规划(2012-2030年)》编制工作，加快中心城区单元、同城化发展节点控规编制，努力实现规划全覆盖。围绕城市拓展和人口集聚需要，积极完善教育、医疗卫生、体育设施和绿地系统、电网、供热布局等专项规划。维护规划严肃性，确保规划实施不走样。
   继续开展城市大建设。全年安排城市大建设项目146个，总投资180亿元。着力实施黄山大道贯通工程，开工建设胜利东路，改善东向交通环境。完成延安路、朝阳路、解放路南延工程，推动城南新区发展。完成解放北路改造，扮靓城市北大门。启动国道104五河段、206怀远段、省道307蚌埠至蒙城一级公路改建，谋划建设省道101长淮卫淮河公路桥及接线工程。推进城市高速公路外环建设，完善城市外环快速通道。开展淮河、浍河、涡河航道整治工程，推进以新港二期集装箱码头为核心的港口群建设，打造皖北内河航运中心。加快京沪高铁蚌埠南站东广场、东站台建设，推进民航机场建设项目前期工作。开工建设7057套保障性住房，实物配租2000户。大力推进征迁拆违，完成市区征迁面积400万平方米，交付净地2万亩，通过征新思路、拆老观念，实现招大项目、引新业态。
   不断加强生态环境建设。落实省政府下达的“千万亩森林增长工程”实施任务，深入开展“绿满珠城”行动和森林城镇、森林村庄建设活动，高起点建设沿天龙河绿化景区带、淮河沿岸景观带、中环线东段防护绿化带，加快张公山公园综合提升改造，建成龙子湖北公园二期，完成黄山大道、水蚌线等地块补绿工程。开工建设陶山植物园，新建、改造老机场三角线、淮河游园等10个街头绿地游园。成片造林12.6万亩，新增改造绿地100万平方米，努力实现“林在路旁、林在村边、林在水岸、林在城中”。做活水文章，启动引河济湖工程，积极推进三汊河湿地公园保护性开发建设，实施淮河、天河湖、张公湖、龙子湖“一河三湖”为主的城市水环境整治。建成运营城南污水处理厂一期。加强城区淮河滩涂环境综合整治，提高绿化、硬化、美化水平。采取生态养鱼和现代渔场新方式，实现全市主要河湖“荡舟无障碍、饮水不担心”。
   持续强化城市长效管理。建设城管数控中心，大力推进城市管理网格化，提高城市管理精细化水平。完善红绿灯、隔离栏、斑马线等交通基础设施，加强客货车超员、超载、超限、疲劳驾驶治理，保障道路交通安全。加大违法设施、违章建筑拆除力度，狠抓占道经营、店面外扩、户外广告、渣土运输等整治，有效解决群众投诉较多的热点问题。加大环卫投入，推进环卫作业机械化、专业化、社会化。
   (六)深化改革、创新、开放，构筑体制机制优势。以改革激发活力、创新引领发展、开放带动跨越，加快形成符合实际、推动发展的体制机制，增强加速崛起的原动力、驱动力、竞争力。
   推进重点领域改革。健全国有资本运营管理机制，完善国有企业经营业绩考核。整合市属国有粮食企业，适时组建粮食企业集团。推进农村金融综合改革，积极创建金融生态县。稳步开展生产经营性事业单位转企改制工作。全面完成县级公立医院综合改革。创新机制，提升104、109等公交线路设施和服务水平。下决心破除阻碍民间投资的“玻璃门”、“弹簧门”等体制障碍，鼓励和支持民营资本投向基础设施和公共事业，进入交通、市政、医疗、环保等领域。继续大力实施“孔雀计划”，在推动已改制企业“二次创业”、支持现有民营企业做大做强的同时，引进更多民营企业，促进民营经济总量上规模、结构上档次、质量上水平。
   增强自主创新能力。突出技术创新主体地位，新增高新技术企业和省级以上创新型企业25家。提升产业创新能力，推进玻璃工业节能技术国家地方联合工程研究中心等研发平台建设，创建大吨位起重机、MEMS等研发中心，新增省级以上研发平台10家。增强创新载体作用，推动蚌埠大学科技园二期建设，谋划建设上海理工大学蚌埠基地二期。加快国家特种玻璃质检中心建设。继续开展企业股权和分红激励政策试点，推进合芜蚌(蚌埠)人才金港建设。
   提升对外开放水平。放大进出口政策效应，支持骨干企业开拓国际市场，进出口总额突破15亿美元。抓好进出口孵化工作，新增进出口经营权备案企业100家。加强口岸基础设施和海关监管场所建设，增强外贸发展服务能力。谋划设立综合保税区，提高口岸开放水平。加快丰原集团、蚌埠玻璃设计院等企业走出去步伐。推动中原经济区三化协调发展先导示范区建设，推进淮河生态经济走廊研究谋划和政策争取。
   (七)注重文明、服务、融合，加快建设文化强市。提升文明水平，彰显文化特色，推进文化大发展大繁荣，以文化凝聚人心、以文化汇集力量、以文化推动跨越。
   培育城市文明新风。加强社会主义核心价值体系建设，广泛开展爱国主义、集体主义、社会主义教育。加大文明创建力度，深入开展市民道德实践活动，放大“好人之城”品牌效应，建立和谐人际关系。大力弘扬城市精神，增强城市凝聚力和市民归属感。深化“三好一满意”活动，大力整治医德医风。全面提升出租车、供电、供气、通信等窗口行业文明服务水平，努力把蚌埠的哥、珠城的姐打造成文明带路人、温馨代名词。加强小区广场、街边游园、城市空地等开放式公共活动场所建设与管理，增加乒乓球台、健身器械等活动设施，活跃百姓大舞台、群众竞技场。
   提升公共文化服务能力。加强双墩、涂山、大禹等与淮河文明密切相关的文化研究、保护、利用，传承创新花鼓灯、泗州戏、五河民歌等地方文化，加强重点文物单位保护。实施文化惠民工程，建成市博物馆、档案馆、规划馆等一批公共文化服务设施，规划和启动奥体中心、大禹博物馆、音乐厅、文化馆、老年和妇女儿童活动场所建设。管理、使用好乡镇综合文化站、农家书屋。开展形式多样、内容丰富的群众文化活动，常年举办各类公益性演出和体育竞赛。
   推动文化产业融合发展。加快文化旅游融合，启动大禹文化产业示范园建设，推进玉文化创意产业园、垓下古战场等项目实施，做大做强玉器古玩、奇石盆景等艺术品业。加快文化教育融合，推进蚌埠儿童电子书与动漫产业园、水木易卡通等项目建设。加快文化科技融合，实施星宇文化创意产业园、“蚌埠兆联天下”科技园区等项目。扶持引导文艺创作生产，推出更多具有淮河文化特色的精品佳作，不断提高蚌埠文化知名度和竞争力。
   (八)聚焦民生、民富、民本，提高群众幸福指数。把群众呼声作为第一信号，把群众需要作为第一选择，把群众满意作为第一标准，致力保障和改善民生，让群众分享更多的发展成果。
   深入实施民生工程。继续抓好33项省级民生工程，注重抓好基本公共卫生服务、县级公立医院药品零差率补助、公共文化场馆开放、农村文化建设专项补助、生活无着人员社会救助、建设美好乡村公共服务体系奖补等6个新增项目。
   各位代表，悠悠万事，民生最大。我们还将从最基础的工作抓起、从最困难的群体帮起，投入2亿元资金，全力实施好12项市级民生工程。
   全线提升公共出行能力：新购置节能环保公交车211辆，新增、延伸、调整公交线路8条，提升改造公交站台200个；改造小街小巷12条，新建、改造路灯4000盏和城市公厕20座。
   全力改善便民生活条件：新建、改造标准化农贸市场6个，提升改造饮食摊点群，完成30家农产品进超市、社区便利店工作；实施市区义务教育阶段公办学校“班班通”，配备多媒体远程教学设备；支持示范社区创建；为市区102个社区添置乒乓球桌；实施“菜篮子”工程，对新建设施蔬菜基地继续奖补。
   全面覆盖扶弱济困措施：建立高龄老人津贴制度，对市区80岁、百岁以上老人每人每年分别补贴300元、2400元；提高对贫困大学生救助标准，每人一次性救助不低于3000元；提高“一户多残”家庭生活补助标准，每户每月补助120元；对困难家庭学龄前儿童入园每人每学期资助600元；对市级民办养老服务机构实行运营补贴，每人每月补贴100元。
   稳定增加城乡居民收入。加快实施居民收入倍增规划，引导广大群众树立通过勤劳致富改善生活的理念，努力增加居民工资性、经营性、财产性、保障性收入。推进创业型城市建设，实施积极的创业就业政策，确保有就业意愿和就业能力的劳动者充分就业。以就读促就业，提高劳动者就业技能，完成职业技能和创业培训3.3万人次，新增城镇就业5万人。
   努力提高社会保障水平。完善社会保障制度，让全市人民不断享受改革发展成果。继续推进社保扩面，稳步提升社会保障标准和待遇水平。扎实开展城乡居民养老保险工作，完善工伤保险配套政策，推进生育保险提标扩面。加快构建以居家为基础、社区为依托、机构养老为支撑的社会养老服务体系。完善社会救助和保障标准与物价上涨挂钩联动机制，着力解决困难群体就学、就业、就医问题。
   (九)致力惠民、便民、安民，维护社会和谐稳定。加强社会建设和管理，加快建立更加完备、更高水准的公共服务和管理体系，努力提高人民群众幸福感和满意度。
   发展各项社会事业。均衡发展义务教育，积极推进素质教育，建设45所幼儿园。加快建设皖北职教园区，二中新校区、市特教中心新校区一期投入使用。建成市疾控中心，开工建设市一院综合住院大楼，推进市二院新院区、三院综合病房楼前期工作。开展与第二军医大学附属长征医院的紧密合作，提升我市医疗技术和医院管理水平，推进建设长征医院蚌埠分院。加快公共体育设施建设，广泛开展全民健身活动，积极申办全国摩托艇大赛、全省中职运动会等国家、省级体育赛事。深入开展拥军优属、拥政爱民活动，扎实推进国防动员和民兵组织建设调整改革。创新与驻蚌单位、大中专院校服务、合作新模式，鼓励科研院所在蚌埠改革转型、加快发展。坚持计划生育基本国策，促进人口长期均衡发展。统筹抓好民族宗教、人防、档案、地方志、外事、侨务、统计、审计、地震、残疾人、妇女儿童、青少年、老龄等工作。
   加强和创新社会管理。完善社区管理体制改革，推行网格化管理服务，实现政府行政管理与基层群众自治有效衔接、良性互动。规范物业管理行为，推行社区监管物业模式。推进政府、中介组织、居民“三位一体”服务体系建设，加强外来人口和特殊人群服务管理。高度重视网络舆情，加强正面引导和安全监管。
   切实维护社会稳定。广泛开展“六五”普法和法律援助，推进依法治市，健全行政执法体制，规范行政执法程序。严厉打击各类违法犯罪活动，构建立体社会治安打防控体系。加强和改进信访工作，着力解决信访热点问题、遗留问题。进一步健全应急管理体系和工作机制，提高保障公共安全和处置突发事件的能力。严格落实安全生产责任制，加强对重点领域、行业、企业和薄弱环节的隐患排查治理，强化食品药品安全监管，确保广大人民群众生命财产安全。
   各位代表，上述九个方面的工作是把握紧迫性、依据可能性，本着务实的态度认真计划安排的，我们担心的是还不能满足广大人民多种层次、不同角度的需求，但我们有决心针对新增热点、难点问题，千方百计争取条件，竭尽全力推动发展，让群众在发展中获得更多的实惠、更大的利益。
   四、凝心聚力，不断锤炼善谋实干的新作风
   各位代表，复杂的经济形势，繁重的发展任务，对新一届政府工作提出了更高的要求。我们将牢记重托，不辱使命，涵养干成事业的志气，鼓足攻坚克难的勇气，练就过硬本领的底气，树立清正廉洁的硬气，努力以更加优良的作风和更大的作为保障和推动科学发展，为人民服务、让人民放心、使人民满意。
   走创新崛起之路，增强创造力。善于乘势而为，敢于逆势而上，各项工作与强的争、同好的比、和快的赛、向高的攀，力争条条战线争上游，以苦干续写蚌埠辉煌，用实干托起蚌埠梦想。坚持把创新作为政府工作的永恒主题和不竭动力，勇于实践、敢于变革、善于创新，用足用活自主创新综合试验区这个大品牌、大载体、大平台，不唯思维定势，不唯发展模式，依据发展实际，大力破除条条框框、惯性思维，积极探索创造跨越发展的新路径、新机制、新模式，让全社会的发展动力、创新活力竞相迸发。
   提科学发展之能，增强战斗力。倍加顾全大局、倍加珍视团结，努力做到心往一处想，劲往一处使，拧成一股绳，团结干事业，齐心谋发展，合力抓落实。深入推进学习型政府建设，在加强学习中充电换脑，在勤于实践中提升水平，切实增强谋划发展、推进工作和破解难题的能力。全面加强公务员队伍建设，打造一支有激情、有本领、有韧劲，善谋事、会干事、能干成事的政府团队。
   倡求真务实之风，增强执行力。围绕育一流作风、强一流效能、创一流业绩的目标，强化“我负责、我来办、马上办”理念，强力推进效能革新。大力精文简会，开短会、发短文、讲短话，腾出更多时间和精力下基层、解难题。处处强化落实，力戒虚夸、浮躁，加大目标考核奖惩力度，加强重大决策、重点工作、重要项目的推进、协调和跟踪督查，坚决杜绝慵散懒，整肃慢作为，惩处不作为、乱作为。
   践为民服务之本，增强亲和力。珠城百姓事，蚌埠公仆情。我们将坚持把群众需求作为引领政府工作的根本导向，把人民满意作为评判政府工作的重要标尺，推行有事帮你忙、问题早解决，让百姓多笑脸，让企业增效益。主动深入一线、走进群众，倾听民声，切实把群众反映最强烈的问题、最不满意的地方、最盼望的事情解决好。认真办好市长热线和政府服务企业热线，实施网上政务服务工程。加大行政审批清理，规范涉企收费，打造零障碍、低成本、高效率的政务环境。
   守清正廉洁之身，增强抵抗力。自觉接受人大法律监督、政协民主监督、新闻舆论监督、网络舆情监督和社会公众监督，完善和加强政府内部监督。重大事项、重大决策及时向人大及其常委会报告、与政协协商，听取各民主党派、工商联、无党派人士及各社会团体的意见。认真办好人大代表议案、建议和政协委员提案。深化政务公开、政府信息公开，建成电子政务一体化平台，推进行政权力网上公开透明运行。全面落实党风廉政建设责任制，继续做好廉政风险防控工作，严格遵守廉洁从政各项规定，进一步增强拒腐防变的自身抵抗能力。大兴艰苦奋斗、厉行节约之风，深入开展政行风评议活动和纠风治乱工作，严肃查办侵犯群众利益的违法违纪案件，做到干部清正、政府清廉、政治清明。
   各位代表，美好的蓝图已经绘就，前进的号角催人奋进。让我们紧密团结在以习近平同志为总书记的党中央周围，在省委、省政府和中共蚌埠市委的坚强领导下，紧紧依靠全市人民，进一步解放思想、开拓创新、攻坚克难，为全面建成小康社会、重返全省第一方阵，早日实现蚌埠复兴之梦而努力奋斗！
